# An Interesting Presentation of Testosterone-Induced Arterial Thrombosis

**DOI:** 10.7759/cureus.14972

**Published:** 2021-05-11

**Authors:** Kevin Thompson, Luis G Osorio, Sami Mughni, Jeffrey Jordan, Olu Oyesanmi

**Affiliations:** 1 Internal Medicine, Citrus Memorial Hospital, Inverness, USA; 2 Rheumatology, Oak Hill Hospital, Brooksville, USA; 3 Internal Medicine, Oak Hill Hospital, Brooksville, USA

**Keywords:** testosterone replacement therapy, thrombosis, arterial thrombosis, testosterone, testosterone-induced thrombosis

## Abstract

Testosterone replacement therapy (TRT) is an industry on the rise in large part due to an increase in direct-to-customer advertising targeting middle-aged men with non-specific symptoms. The biggest problem with unnecessary prescribing is that testosterone therapy is not without side effects. One of the more common adverse effects is erythrocytosis with subsequent thrombosis. It was originally postulated that thrombosis seen in patients on TRT was solely related to increasing in hemoglobin however, new studies demonstrate increasing episodes of thrombosis unrelated to hemoglobin or hematocrit.

We report the case of a 38-year-old white male presenting to the clinic with infarction of bilateral feet and digits due to testosterone-induced thrombosis of dermal and epidermal arteries. Laboratory workup including vasculitis panel was negative and complete blood count (CBC) was within appropriate parameters. He was treated with anticoagulation, pain control, and vasodilatory therapy with subsequent improvement of symptoms.

There have been many reported cases of testosterone-induced thrombosis of the venous system with occasional involvement of the renal arteries. However, cases involving thrombosis of dermal or epidermal arteries due to testosterone supplementation have never been reported. It could be beneficial to screen potential patients requiring TRT for hypercoagulable states such as Factor V Leiden and lupus anticoagulant.

## Introduction

Testosterone therapy has been the mainstay of treatment for hypogonadism according to the Endocrine Society’s Clinical Practice Guidelines [1]. Testosterone may help improve sexual desire, erectile function, and sexual satisfaction [2]. However, like most medications, exogenous testosterone use does not come without risks, this same systematic review showed an increased risk of erythrocytosis, as well as lower urinary tract symptoms [2].

A 2020 meta-analysis of randomized controlled trials by Ayele et al. showed that there was no increased risk of venous thromboembolism (VTE) with testosterone replacement therapy (TRT) however, a clinically increased risk was not ruled out [3]. This increased risk comes in the form of a previous history of thrombophilia-hypofibrinolysis. A history of Factor V Leiden (FVL), lupus anticoagulant, and high lipoprotein (a) was found to significantly increase the risk of a VTE event and recurrence of VTE [4]. VTE pathogenesis is multifactorial. VTE can be serious, and sometimes fatal with 600,000 hospitalizations and 60,000 deaths in the USA alone [5]. The risk factors of VTE and arterial thrombus often overlap, and having risk factors for VTE has been shown to have an increased risk of arterial thrombosis as well [6].

We present the case of a patient with a history of deep venous thrombosis (DVT) and pulmonary embolism (PE) while on testosterone presenting with a vasculitis-like appearance of the bilateral lower extremity after starting testosterone therapy once again. To our knowledge, current literature has not shown a case of testosterone-induced thrombosis of small vessels leading to epidermal and dermal ischemia.

## Case presentation

We report the case of a 38-year-old white male with a history of DVT and PE nine years ago, who presented to the clinic with discoloration and pain of bilateral feet and lower extremity digits. The patient reported that his symptoms began with tingling in the toes and later numbness. A week after his initial symptoms he described the feeling of severe pain in his feet. At this time he started to notice a change in the coloration of his toes bilaterally. He described them as becoming dark purple and appearing blotchy in certain areas, with blanching on palpation. The patient stated the pain in his feet and toes was typically more severe at night. The pain became so severe that it began to interfere with his work at a construction company. He had been experiencing these issues for about a month and it has been slowly worsening over time. He has never experienced manifestations like those before. The only medication he had been taking was testosterone cypionate 250 mg 1 cc per week, which he began five to six months ago. He said he received this prescription from a men’s health clinic after voicing symptoms of low energy and loss of libido. His laboratory results revealed borderline low testosterone levels, 340 ng/dl. After further questioning, the patient admitted that this was not his first time receiving testosterone therapy. He reported that around nine years ago, he received testosterone and trenbolone (an anabolic steroid traditionally used on livestock) to increase muscle mass from a friend at his gym. At this time he was taking thrice the dosage as he was now. He stopped supplementation after five months when he was diagnosed with DVT as well as PE which led to hospitalization. The symptoms during this presentation differed significantly from those experienced during his hospitalization from DVT/PE. The blotchy and purple discoloration of his digits and feet which blanched with pressure appeared similar to palpable purpura or embolic disease.

The patient initially presented to his primary care physician (PCP) who started him on rivaroxaban 10 mg daily and after minimal symptomatic improvement, a rheumatological evaluation was requested, due to vasculitis concern. Workup included a 2D echocardiogram which was negative for thrombus or vegetation. Complete blood count (CBC), comprehensive metabolic panel (CMP), testosterone levels, prolactin, erythrocyte sedimentation rate (ESR), and C-reactive protein were within normal parameters. Anti-nuclear antibody (ANA), anti-neutrophil cytoplasmic antibody (ANCA), and hepatitis viral panel were negative. Ankle-brachial indices demonstrated normal results as well. The patient was clinically diagnosed with bilateral lower extremity ischemia with infarction secondary to testosterone therapy. The most likely pathogenesis behind this case is testosterone-induced hypercoagulability with ensuing dermal and epidermal vascular occlusion with infarction.

During the previous admission, thromboses involved the venous system, subsequent involvement of the arterial system suggests that testosterone-induced hypercoagulability confers risk for both venous and arterial thrombosis. His treatment regimen consisted of rivaroxaban 10 mg daily, full dose aspirin (325 mg) daily, amlodipine 10 mg daily, and sildenafil 10 mg daily to prevent blood clots and improve blood flow to ischemic regions. Neuropathic pain control with gabapentin 100 mg at bedtime and pain control with tramadol 25 mg twice a day as needed. Most importantly, the patient was advised to discontinue the use of testosterone therapy. He reported improvement in symptoms within 2-3 days of beginning a full treatment regimen.

## Discussion

TRT is an industry on the rise in large part due to increasing direct-to-customer advertising, which frequently targets middle-aged men with non-specific symptoms such as fatigue and decreased libido [7]. One of the biggest problems with the increase in unnecessary testosterone supplementation is that this therapy is not without potential side effects. This was evident in our patient who presented with ischemia and infarction of his bilateral lower extremities. A common side effect of testosterone is erythrocytosis, which was initially believed to be the sole source of thrombosis formation seen in these patients [2]. Per Braekkan et al., thrombosis formation is directly related to hematocrit [8]. Nevertheless, in many instances patients with DVT and PE while receiving testosterone supplementation did not present with erythrocytosis. In a study (N = 42) that evaluated the presence of DVT and PE in patients on testosterone which was unrelated to erythrocytosis, 27 had DVT or PE, 12 with osteonecrosis, one with amaurosis fugax, one with central retinal vein occlusion, and one with spinal cord infarction [4]. Osteonecrosis was included because the necrosis of the femur in these patients was believed to be due to clot formation in the veins of the femur head. According to the study, patients on testosterone therapy only experienced thromboses of the venous system, yet in our case, the site of thrombosis was the arterial small vessels.

Although arterial thromboses secondary to testosterone therapy are not commonly reported in the literature, a small number of cases do exist. An example of arterial thrombosis was reported in a 43-year-old white male with a history of obsessive-compulsive disorder presenting with flank pain [9]. The patient underwent contrast computerized tomography angiography (CTA) of his abdomen and pelvis which revealed a renal infarction. He was found to have thrombosis of his renal artery. This patient later admitted to using trenbolone and testosterone for a period of five years and was treated with heparin drip and later transitioned to warfarin therapy [9].

Another case of arterial occlusion was seen in a 25-year-old white male bodybuilder who presented with chest pain [10] He had an electrocardiogram which revealed diffuse ST elevation. Troponins were taken which showed elevated levels >50 ng/mL. Emergent percutaneous coronary intervention demonstrated a large occlusive thrombus in the left anterior descending artery. This was treated with aspiration thrombectomy and stent placement. The patient also acknowledged years of intramuscular testosterone use. He was discharged on aspirin and clopidogrel. Several days later he returned to the hospital with abdominal pain, later to be diagnosed by CTA with renal artery thrombosis. He received treatment with enoxaparin and was bridged to warfarin.

In our case report, the patient presented with pain and discoloration of his bilateral lower extremities and digits. His workup for an inflammatory pathology as well as cardiogenic embolus was negative. Clinical diagnosis of bilateral lower extremity ischemia and infarction due to testosterone therapy was made. He then received appropriate treatment with pain control, anticoagulation, and vasodilation. Although testosterone-associated venous, coronary artery, and renal artery thrombosis have been frequently described, there have been no cases reported in the literature of small vessel arterial thrombus formation resulting in dermal and epidermal infarction, as seen in our case report.

## Conclusions

Avoiding the use of TRT in patients with a previous history of VTE is an important consideration for every physician to be aware of. Evidence-based recommendations should be updated to illustrate the need for screening tests for conditions such as FVL, lupus anticoagulant, increased lipoprotein (a) in a similar manner as done when starting oral contraceptives in female patients.
